# Mendelian Randomization Analysis Suggests No Associations of Herpes Simplex Virus Infections With Multiple Sclerosis

**DOI:** 10.3389/fnins.2022.817067

**Published:** 2022-03-01

**Authors:** Wan Zhang, Pengfei Wu, Rui Yin, Meichen Sun, Rongsen Zhang, Xiaoyao Liao, Yuhong Lin, Hui Lu

**Affiliations:** ^1^Department of Biology, Boston University, Boston, MA, United States; ^2^Department of Neurology, Beth Israel Deaconess Medical Center, Boston, MA, United States; ^3^School of Life Sciences, Central South University, Changsha, China; ^4^Department of Biomedical Informatics, Harvard Medical School, Boston, MA, United States; ^5^Department of Neurology, Xuanwu Hospital, Capital Medical University, Beijing, China; ^6^Department of Ultrasound, Second Xiangya Hospital, Central South University, Changsha, China; ^7^College of Medical Veterinary and Life Sciences, University of Glasgow, Glasgow, United Kingdom; ^8^Zhongshan School of Medicine, Sun Yat-sen University, Guangzhou, China

**Keywords:** multiple sclerosis, herpes simplex virus, genetic epidemiology, Mendelian randomization, causal risk factors

## Abstract

Previous studies have suggested an association between infection with herpes simplex virus (HSV) and liability to multiple sclerosis (MS), but it remains largely unknown whether the effect is causal. We performed a two-sample Mendelian randomization (MR) study to explore the relationship between genetically predicted HSV infection and MS risk. Genetic instrumental variables for diagnosed infections with HSV (*p* < 5 × 10^–6^) were retrieved from the FinnGen study, and single nucleotide polymorphisms associated with circulating immunoglobulin G (IgG) levels of HSV-1 and HSV-2 and corresponding summary-level statistics of MS were obtained from genome-wide association studies of the European-ancestry. Inverse-variance weighted MR was employed as the primary method and multiple sensitivity analyses were performed. Genetically proxied infection with HSV was not associated with the risk of MS (odds ratio [OR], 0.96; 95% confidence interval [CI], 0.90–1.02; *p* = 0.22) per one-unit increase in log-OR of herpes viral infections. MR results provided no evidence for the relationship between circulating HSV-1 IgG levels and MS risks (OR = 0.91; 95% CI, 0.81–1.03; *p* = 0.37), and suggested no causal effect of HSV-2 IgG (OR = 1.04; 95% CI, 0.96–1.13; *p* = 0.32). Additional sensitivity analyses confirmed the robustness of these null findings. The MR study did not support the causal relationship between genetic susceptibly to HSV and MS in the European population. Further studies are still warranted to provide informative knowledge, and triangulating evidence across multiple lines of evidence are necessary to plan interventions for the treatment and prevention of MS.

## Introduction

Multiple sclerosis (MS) is the most common chronic demyelinating and neurodegenerative disease of the central nervous system (CNS) (Hauser and Cree, 2020). It is the leading cause of non-traumatic neurological disability in young adults, affecting more than 2 million people worldwide (GBD, 2017). The symptoms of MS usually follow relapsing or progressive path, eventually leading to impaired mobility or cognition (Reich et al., 2018). MS is currently incurable though therapeutic advances have remarkably improved the long-term outcome for patients at this time (Hauser and Cree, 2020; Iqubal et al., 2020). The etiology of MS has not been fully elucidated. Early infections with herpes simplex virus (HSV) infection are constantly proposed to be involved in the pathogenesis of MS. HSV-1 and HSV-2 infections usually occur in the early years of life, mostly latent and asymptomatic (Koyuncu et al., 2013). HSV viruses lurk in the sensory ganglion of the trigeminal nerve, remain exist lifelong, and could invade CNS (Kimberlin et al., 2001). Post-mortem results have also confirmed the presence of HSV in brain demyelinating plaques of MS patients (Sanders et al., 1996).

Based on retrospective data in Sarajevo, the positive incidence of HSV immunoglobulin G (IgG) antibodies was 93.2% in 110 newly diagnosed MS patients (Djelilovic-Vranic and Alajbegovic, 2012). In another study, the prevalence of HSV-1 mRNA and DNA in the peripheral blood mononuclear cells (PBMC) of acute MS patients is significantly higher compared to controls (Ferrante et al., 2000). They also suggested that HSV-1 reactivate in the acute attack and might trigger MS relapses (Ferrante et al., 2000). Data addressing pediatric MS showed that HSV-1 IgG antibodies in serum was associated with increased risk of pediatric MS (Waubant et al., 2011; Nourbakhsh et al., 2018). Waubant et al. (2011) recruited 189 pediatric MS patients and found that HSV-1 was associated with an increased risk of MS in those negative for HLA DRB1*1501. Another multi-center research suggested that sero-positivity for HSV-1 was significantly increased in pediatric MS patients, but the increase was only seen in Caucasian people and those without a DRB1*15 allele (Nourbakhsh et al., 2018). Pooled results of a recent meta-analysis has implicated a statistical difference in the serum prevalence of IgG against HSV-2 between patients with MS and controls (Xu et al., 2021).

However, other studies reported conflicting results, and did not find any relationship between HSV infection and MS risk. Data in several studies showed that the prevalence of antibodies against HSV-1 or HSV-2 had no statistical associations with adult MS (Wandinger et al., 2000; Kiriyama et al., 2010; Sotelo et al., 2014; Etemadifar et al., 2019). By testing HSV DNA in cerebrospinal fluid or in PBMC, Koros et al. (2014) and Sotelo et al. (2014) reported no significant difference of HSV DNA between adult MS and healthy controls. Another pediatric study found no difference in the association of prior HSV infections with the onset of pediatric MS (Mowry et al., 2011).

Those equivocal results might be caused by methodological shortcomings of observational studies, such as residual confounding and reverse causality. Confined by these limitations, observational research is unable to deduce the causal role of HSV infection in the development of MS. With the exponential growth in and widespread availability of genotype data, Mendelian randomization (MR) approach as an epidemiologic study designed to establish causality between exposures and outcomes has gained its popularity in the last two decades (Zhuang et al., 2019; Huang et al., 2021; Kwok and Schooling, 2021; Zhang et al., 2021). MR utilizes germline genetic variants as proxies. Since genetic variants are unaffected by environmental factors or disease process, MR can diminish confounding, strengthen exposure-outcome associations and avoid reverse causalities (Smith and Ebrahim, 2003). In this study, we leveraged the MR approach to infer the associations of HSV infection with risk of MS.

## Materials and Methods

**FIGURE 1:**
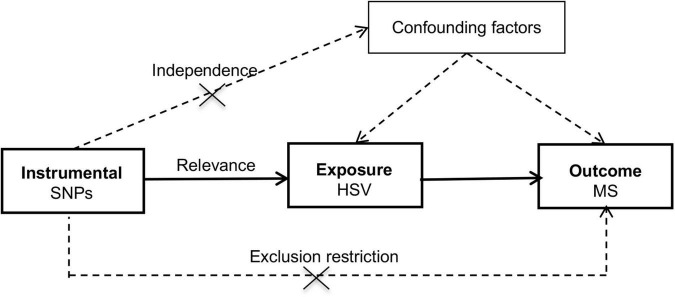
Schematic diagram of the Mendelian randomization study. HSV, herpes simplex virus; MR, Mendelian randomization; MS, multiple sclerosis; SNP, single nucleotide polymorphism.

The schematic for the MR design was shown in Figure 1 and datasets underlying the study was summarized in Supplementary Table 1. This study was built upon summary-level statistics which were publicly accessible. Informed consent from participants and approval by ethical committees had been completed by consortia involved in original studies.

### Instrumental Variables for Herpes Simplex Virus

Genetic instrumental variants for HSV infection were obtained from the FinnGen study (FinnGen, 2021). Diagnosed infections with HSV were defined by International Classification of Diseases (ICD) from the Finnish registries of inpatient, outpatient and cause of death. In the R5 release, there were 1,595 cases (ICD-10, B00; ICD-9, and ICD-8, 054) and 211,856 participating controls of Finnish ancestry. Sex, age, 10 principal components and genotyping batch were included as logistic regression covariates. Eight instrumental single-nucleotide polymorphisms (SNPs) were selected at a suggestive genome-wide significance threshold (*p* < 5 × 10^–6^) as previous studies did (Kodali et al., 2018; Bae and Lee, 2020; Kwok and Schooling, 2021). The effect size was presented in a unit of log- odds ratio (OR) using the additive model (Supplementary Table 2).

Instrumental variables for circulating HSV-1 and HSV-2 IgG levels were selected from one genome-wide association study (GWAS) conducted in the Milieu Intérieur cohort (Scepanovic et al., 2018). Total IgG levels and antigen specific seropositivity was tested in 1,000 individuals, and in seropositive donors, serum IgGs specific for HSV-1 (*n* = 645) and HSV-2 (*n* = 208) were further measured using the BioPlex™ 2200 HSV-1 and HSV-2 IgG kit (Bio-Rad, Hercules, CA, United States). After log10-transformed of IgG levels, genetic association analyses were performed with the additive regression adjusted for age, sex, total IgG and the first two principal components incorporated as covariates. Four and eight SNPs were utilized as instrumental variables for HSV-1 IgG (Supplementary Table 3) and HSV-2 IgG (Supplementary Table 4), respectively.

### Summary-Level Datasets of Multiple Sclerosis

Summary-level GWAS results of MS were released by the International Multiple Sclerosis Genetics Consortium (2019). In total, 14,802 individuals diagnosed with MS and 26,703 healthy controls of the European-ancestry were recruited in the discovery stage with 8,589,719 SNPs being meta-analyzed. Diagnostic criteria and demographic descriptions in each contributing cohort were summarized in the published GWAS (International Multiple Sclerosis Genetics Consortium, 2019). Effect estimates were adjusted for age, sex, batch effects and ten principal components in the logistic regression, and *Beta* represented one-unit increase in log-OR of MS per additional effect allele (Supplementary Table 5). Here, the following formulae were employed in transforming variables: *Beta* = *log*(*OR*) and *Standarderror* = *Beta*/*abs*((*qnorm*(*P*−*value*/2)). We kept instrumental SNPs which were present in the MS dataset, or whose proxied SNPs (*r*^2^ > 0.8 or *D*’ > 0.8, EUR panel 1000 Genomes Phase 3) were available. We harmonized the exposure and outcome effect size in terms of the effect allele and merged datasets were used for subsequent analyses.

### Statistical Analysis

We performed MR analyses in the R language, version 3.6.1 (R Foundation for Statistical Computing, Vienna, Austria) with the TwoSampleMR and MR-PRESSO packages (Hemani et al., 2018; Verbanck et al., 2018). Effect of HSV related exposures on the risk of MS contributed by individual instrumental variable was first given by Wald ratio: *Y*_*k*_/*X*_*k*_ with its standard error óó*Y*_*k*_/*X*_*k*_, where the SNP-effect on HSV was denoted with *X_k_* and its standard error σ*X*_*k*_, and the SNP-MS association statistics denoted with *Y_k_* and σ*Y*_*k*_. Then the primary MR method, the inverse-variance weighted (IVW) model combined ratio estimates for each exposure and yielded an overall estimate: ${\hat{\beta }}_{M\,R}=\sum X_{k}\,Y_{k}\,\sigma_{Y_{k}}^{-2}/\sum X_{k}^{2}\,\sigma_{Y_{k}}^{-2}$ with ${\hat{\sigma }}_{M\,R}=\sqrt{1/\sum X_{k}^{2}\,\sigma_{Y_{k}}^{-2}}$. Based on stringent prerequisites, IVW estimates would be biased if not all variants are valid or unbalanced pleiotropy exists (Burgess et al., 2013). Three additional approaches were implemented. Weighted median method effectively pooled individual estimate if less than half instrumental SNPs were invalid (Bowden et al., 2016). MR-Egger regression identified horizontal pleiotropic effects with *p* for intercept <0.05, meanwhile the regression slope provided a causal estimate corrected for unbalanced pleiotropy (Bowden et al., 2015). MR-PRESSO also examined outlier SNPs with potential pleiotropy by the global test and computed both a raw estimate and an outlier-adjusted estimate (Verbanck et al., 2018). We conducted Cochran’s *Q* test and leave-one-out analysis to identify individual SNP which exerted an extremely heterogenous effect. As a measure of causal associations between HSV-related exposures and the risk of MS, we reported OR and 95% confidence interval (CI) per one unit increase in log-OR of diagnosed HSV infection or one SD elevation in circulating IgG levels of HSV-1 or HSV-2. Associations with *P* < 0.05/3, using the Bonferroni correction, were deemed as significant.

## Results

### Association of Herpes Simplex Virus Infection With Multiple Sclerosis Risk

**FIGURE 2:**
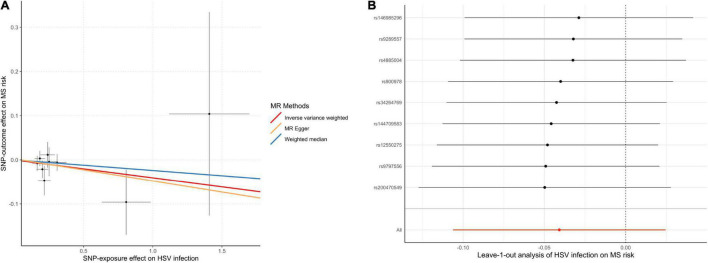
Scatter plot **(A)** and leave-one-out plot **(B)** in the Mendelian randomization analysis of HSV infection on MS risk. HSV, herpes simplex virus; MR, Mendelian randomization; MS, multiple sclerosis.

In the MR analysis investigating the relationship between infections with HSV and MS risk, nine instrumental SNPs were utilized and they collectively explained 0.09% variances of HSV (Supplementary Table 2). MR results suggested that diagnosed infections with HSV were not associated with the risk of MS (Table 1). By the IVW method, OR of MS was 0.96 (95% CI, 0.90–1.02; *p* = 0.22) per one-unit increase in log-OR of herpes viral infections. Sensitivity analyses by weighted median, MR-Egger regression slope and MR-PRESSO provided similar and consistent results. There was no evidence of pleiotropy by MR-Egger regression intercept (*p* = 0.85) or MR-PRESSO global test (*p* = 0.91). Besides, Cochran’s *Q* test (Table 2) and leave-one-out analysis (Figure 2) indicated no heterogeneity among the instrumental SNPs.

**TABLE 1:** Association of genetically predicted herpes simplex virus infection with the risk of multiple sclerosis by different Mendelian randomization approaches.

MR methods	HSV infection			HSV-1 IgG			HSV-2 IgG		
	OR	95% CI	*P*-value	OR	95% CI	*P*-value	OR	95% CI	*P*-value
Inverse variance weighted	0.96	0.90–1.02	0.22	0.75	0.35–1.60	0.45	1.04	0.96–1.13	0.32
Weighted median	0.98	0.90–1.06	0.58	0.92	0.67–1.27	0.62	1.03	0.94–1.14	0.49
MR-Egger regression slope	0.95	0.80–1.13	0.59	0.45	0.004–44.82	0.76	0.89	0.58–1.36	0.60
MR-PRESSO raw estimate	0.96	0.92–1.01	0.12	0.75	0.35–1.60	0.51	1.04	0.96–1.13	0.35
MR-PRESSO outlier corrected	–	–	–	0.91	0.81–1.03	0.37	–	–	–

*CI, confidence interval; HSV, herpes simplex virus; MR, Mendelian randomization; MR-PRESSO, Mendelian randomization pleiotropy residual sum and outlier; OR, odds ratio.*

**TABLE 2:** Results from Mendelian randomization sensitivity analyses between herpes simplex virus (HSV) and multiple sclerosis (MS).

Exposures	MR-Egger regression			Heterogeneity test		MR-PRESSO global test	
	Intercept	SE	*P*-value	*Q* statistic	*P*-value	*RSSobs*	*P*-value
HSV infection	0.002	0.02	0.91	4.05	0.85	4.92	0.88
HSV-1 IgG	0.05	0.21	0.84	33.98	<0.001	58.55	<0.001
HSV-1 IgG (excluding rs3132935)	0.04	0.11	0.79	5.06	0.08	–	–
HSV-2 IgG	0.03	0.04	0.47	8.77	0.27	11.42	0.30

*MR-PRESSO, Mendelian randomization pleiotropy residual sum and outlier; RSSobs, observed residual sum of squares; SE, standard error. MR-PRESSO global test was not available when examining the association of HSV-IgG (excluding rs3132935) with multiple sclerosis due to insufficient number of genetic instrumental variables.*

### Effect of Circulating Herpes Simplex Virus-1 and Herpes Simplex Virus-2 Immunoglobulin G Levels on Multiple Sclerosis Risk

**FIGURE 3:**
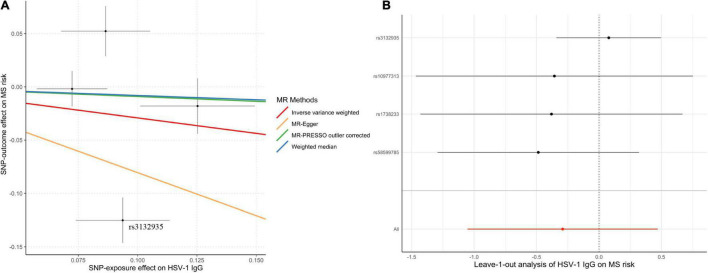
Scatter plot **(A)** and leave-one-out plot **(B)** in the Mendelian randomization analysis of HSV-1 IgG levels on multiple sclerosis. MR-PRESSO outlier-corrected estimate was calculated with the removal of rs3132935 as an outlying variant, while the raw estimate was not delineated since it was nearly the same as the value given by inverse-variance-weighted method. HSV, herpes simplex virus; MR, Mendelian randomization; MR-PRESSO, Mendelian randomization pleiotropy residual sum and outlier; MS, multiple sclerosis.

Genetically predicted HSV-1 IgG was not associated with the risk of MS (OR = 0.75; 95% CI, 0.35–1.60; *p* = 0.45) by the IVW method. Notably, rs3132935 was associated with MS at genome-wide significance (*p* = 3.40 × 10^–9^). MR-PRESSO global test, Cochran’s *Q* test (Table 2) and leave-one-out analysis (Figure 3) all indicated that rs3132935 might have pleiotropic effects and was an outlier variant in the MR analysis. Nevertheless, the MR-PRESSO corrected estimate with the removal of rs3132935 suggested no causal effect of circulating HSV-1 IgG levels on MS risks (OR = 0.91; 95% CI, 0.81–1.03; *p* = 0.37), either.

**FIGURE 4:**
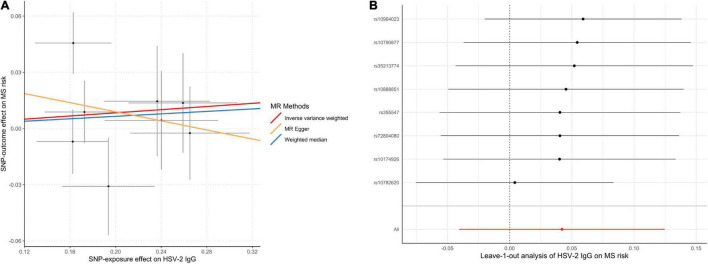
Scatter plot **(A)** and leave-one-out plot **(B)** in the Mendelian randomization analysis of HSV-2 IgG on MS risk. HSV, herpes simplex virus; MR, Mendelian randomization; MS, multiple sclerosis.

The MR analyses did not support the causal effect of HSV-2 IgG on MS (OR = 1.04; 95% CI, 0.96–1.13; *p* = 0.32) per one SD increase in HSV-2 IgG levels. Additional MR methods provided consistent results (Table 1). Furthermore, no unbalanced horizontal pleiotropy or evident heterogeneity was identified through multiple sensitivity analysis (Figure 4).

## Discussion

Seroprevalence of HSV is ubiquitous in populations where MS is prevalent (50–100% in adult members) (Lycke, 2017). In response to exposure to HSV-1, persistent lymphocytic cells would infiltrate in the CNS, levels of cytokine transcripts would elevate, and amounts of chemokine mRNAs would increase, which suggest that latent HSV-1 infection might trigger a chronic inflammatory process in brain tissue (Theil et al., 2003; Menendez et al., 2016). Meanwhile, levels of matrix metalloproteinases 2 and 9 would increase in infected CNS, and contribute to a partial breakdown of the blood brain barrier which is crucial in MS (Martínez-Torres et al., 2004). After exposure to HSV-1, plasmacytoid dendritic cells (DC) produce a great deal of Type I IFNs, including IFN-α and β (Soumelis and Liu, 2006). Plasmacytoid DC can promote naïve T cells to produce IL-10, which leads to anti-inflammatory reactions (Rissoan et al., 1999). Type I IFNs can inhibit the production of IL-12 and increase IL-10 production, which act on myeloid DC and switch pro- to anti-inflammation (Sanna et al., 2008). PBMC of MS patients showed increased production of IFN-α, IL-6, and IL-10 but decreased production of IL-4 (Sanna et al., 2008) and productions of IL-6 and IL-10 by PBMC and plasmacytoid DC were lower in MS patients compared with healthy controls (Sanna et al., 2008). The above data implied impaired anti-inflammatory response after HSV-1 infection in MS. Animal experiments have further proved that previous exposure to HSV-1 can cause an earlier onset of symptoms and more severe experimental autoimmune encephalomyelitis compared to uninfected control mice (Duarte et al., 2021).

Clinical trials of antiviral treatments in MS (Lycke et al., 1996; Bech et al., 2002; Friedman et al., 2005) were limited when compared with the development of other therapies (Lizak et al., 2017; Islam et al., 2020). There are three phase II clinical trials of acyclovir or valacyclovir in MS patients (Lycke et al., 1996; Bech et al., 2002; Friedman et al., 2005). One trial showed 34% reduction of annualized relapse rate in acyclovir-treated patients and a significant reduction in the relapse rate in favor of acyclovir treatment (Bech et al., 2002). In a high-activity group of another trial, valacyclovir-treated patients had significant reduction of new lesions compared to placebo-treated patients (Friedman et al., 2005). Although the above research suspected HSV as a candidate for the etiology of MS, the fact that HSV infection is far more prevalent in human populations compared to MS argues against this viewpoint. HSV DNA in 77 demyelinated plaques from 23 MS patients revealed that HSV-1 DNA was amplified from only one plaque and HSV-2 DNA was amplified from none of the plaques (Nicoll et al., 1992). The infection of HSV in the CNS might be insufficient for the development of MS which requires other genetic and environmental triggers. Further investigations are warranted to detangle the role of HSV in disease onset or disease progression of MS.

The major strength of this study is the multivariable MR method, which explicated the roles of HSV infection in MS and exempted the result from residual confounding or reverse causality. Also, up-to-date genetic instruments for HSV infection traits and the largest GWAS dataset for MS were used to boost the power. There are several limitations for this study. Firstly, instrumental SNPs collectively explained small proportions of variance for HSV infection, and especially for circulating IgG levels of HSV-1 and HSV-2 due to inadequate sample size. Hence, we had restricted power to identify small causal effects. Secondly, we used a relaxed significance level (*p* < 5 × 10^–6^) rather than the classical GWAS threshold (*p* < 5 × 10^–8^) to choose instrumental variables. Distortion to the overall estimate might occur in the scenario, albeit no weak instrument was identified in the present study. Thirdly, biological implications for most SNPs are yet to be explored; thus, the suitability of current instrumental sets would be disputed by the possibility of pleiotropy, although no pleiotropic effects (except for rs3132935) were indicated through our sensitivity analyses. Lastly, this study was based on genome-wide association data only from the Europeans and we should be cautious with the interpretation and generalization when it comes to other populations.

## Conclusion

In conclusion, we failed to provide evidence for the effect of HSV on the risk of MS. Further studies triangulating evidence from observational cohorts, clinical trials and genetic-epidemiological biobanks are still warranted to elucidate whether targeting HSV is an effective intervention for MS.

## Data Availability Statement

The original contributions presented in the study are included in the article/Supplementary Material, further inquiries can be directed to the corresponding author.

## Author Contributions

WZ, PW, and HL conceptualized the study. PW, RY, RZ, and HL contributed to the data analysis and interpretation. WZ, MS, XL, and YL contributed to the manuscript drafting and editing. All authors contributed to the article and approved the submitted version.

## Conflict of Interest

The authors declare that the research was conducted in the absence of any commercial or financial relationships that could be construed as a potential conflict of interest.

## Publisher’s Note

All claims expressed in this article are solely those of the authors and do not necessarily represent those of their affiliated organizations, or those of the publisher, the editors and the reviewers. Any product that may be evaluated in this article, or claim that may be made by its manufacturer, is not guaranteed or endorsed by the publisher.
