# Supplementary material for: Mendelian Randomization Analysis Suggests No Associations of Herpes Simplex Virus Infections With Multiple Sclerosis
Source: Front Neurosci. 2022 Mar 1;16:817067. doi: 10.3389/fnins.2022.817067 (PMC8920987; doi:10.3389/fnins.2022.817067)
Supplement: Supplementary file 1 [file Data_Sheet_1.PDF]

Supplementary Material

Supplementary Table 1. Brief description of datasets utilized in the Mendelian randomization study

Supplementary Table 2. Instrumental variables for genetically predicted HSV infection in the Mendelian randomization analysis

Supplementary Table 3. Instrumental variables for genetically predicted HSV-1 IgG levels in the Mendelian randomization analysis

Supplementary Table 4. Instrumental variables for genetically predicted HSV-2 IgG levels in the Mendelian randomization analysis

Supplementary Table 5. Summary-level statistics from the genome-wide association study of multiple sclerosis

Supplementary Table 1. Brief description of datasets utilized in the Mendelian randomization study

Exposure and Outcome	Source	Sample size	Cases	Controls	Population	Access Link
HSV infection	FinnGen (R5)	213,451	1595	211,856	European	https://r5.finngen.fi/
HSV-1 IgG	Milieu Intérieur cohort	645	—	645	European	https://doi.org/10.5281/zenodo.1217136
HSV-2 IgG	Milieu Intérieur cohort	208	—	208	European	https://doi.org/10.5281/zenodo.1217136
Multiple sclerosis	IMSGC	41,505	14,802	26,703	European	https://imsgc.net

Abbreviations: HSV, herpes simplex infection; IMSGC, International Multiple Sclerosis Genetics Consortium.

Supplementary Table 2. Instrumental variables for genetically predicted HSV infection in the Mendelian randomization analysis

SNP	CHR	Position (GRCh37)	Nearest Gene	Effect allele	Other allele	EAF	Association with diagnosed HSV infection			R^2 (%)	*F*-statistic
							Beta	SE	*P*-value		
rs144709583	1	165,967,856	FAM78B	T	C	0.01	1.41	0.29	1.26×10^{-6}	0.0110	23.49
rs900978	2	193,321,821	TMEFF2	T	C	0.45	0.16	0.04	4.62×10^{-6}	0.0098	20.96
rs9289557	3	138,071,604	MRAS	T	C	0.19	0.22	0.05	2.37×10^{-6}	0.0104	22.30
rs146985296	6	52,152,875	MCM3	C	G	0.01	0.81	0.18	4.68×10^{-6}	0.0098	20.97
rs34264769	8	2,639,919	CSMD1	A	C	0.15	0.25	0.05	1.38×10^{-6}	0.0109	23.33
rs12550275	8	68,909,451	PREX2	T	C	0.14	0.24	0.05	4.84×10^{-6}	0.0098	20.87
rs4885004	13	73,093,913	MZT1	G	A	0.22	0.20	0.04	3.23×10^{-6}	0.0101	21.64
rs9797556	19	46,500,735	CCDC61	G	A	0.33	0.18	0.04	1.41×10^{-6}	0.0109	23.25
rs200470549	22	50,548,270	MOV10L1	TA	T	0.08	0.31	0.07	4.71×10^{-6}	0.0098	20.96

Abbreviations: SNP, single nucleotide polymorphism; CHR, chromosome; EAF, effect allele frequency; SE, standard error.

Notes: Proportion of variance explained by individual SNP was calculated as $R^2 = 2*\beta^2*EAF*(1-EAF)/(2*\beta^2*EAF*(1-EAF) + 2*SE^2*N*EAF*(1-EAF))$, where sample size was denoted by N = 213,451 (including 1595 cases and 211,856 controls from the FinnGen Biobank); *F*-statistic was computed using the formula $F = R^2*(N-2)/(1-R^2)$, where $F < 10$ indicated a weak instrument variant. The aforementioned equations were given by H. Shim, *et al., PLoS One* **10**, e0120758 (2015).

Supplementary Table 3. Instrumental variables for genetically predicted HSV-1 IgG levels in the Mendelian randomization analysis

SNP	CHR	Position (GRCh37)	Nearest Gene	Effect allele	Other allele	EAF	Association with HSV-1 IgG			R^2 (%)	*F*-statistic
							Beta	SE	*P*-value		
rs3132935	6	32,171,075	NOTCH4	G	A	0.17	0.09	0.02	2.76×10^{-6}	3.37	22.42
rs1738233	6	38,766,488	DNAH8	G	A	0.57	-0.07	0.01	1.45×10^{-6}	3.56	23.71
rs58599785	6	139,865,470	LOC645434	G	A	0.17	0.09	0.02	4.91×10^{-6}	3.20	21.28
rs10977313	9	8,828,849	PTPRD	T	C	0.11	-0.13	0.02	2.97×10^{-7}	4.02	26.91

Abbreviations: SNP, single nucleotide polymorphism; CHR, chromosome; EAF, effect allele frequency; SE, standard error.

Notes: Proportion of variance explained by individual SNP was calculated as $R^2 = 2*\beta^2*EAF*(1-EAF)/(2*\beta^2*EAF*(1-EAF) + 2*SE^2*N*EAF*(1-EAF))$, where sample size was denoted by N = 645 (seropositive individuals recruited in the *Milieu Intérieur* cohort); *F*-statistic was computed using the formula $F = R^2*(N-2)/(1-R^2)$, where $F < 10$ indicated a weak instrument variant. The aforementioned equations were given by H. Shim, *et al., PLoS One* **10**, e0120758 (2015).

Supplementary Table 4. Instrumental variables for genetically predicted HSV-2 IgG levels in the Mendelian randomization analysis

SNP	CHR	Position (GRCh37)	Nearest Gene	Effect allele	Other allele	EAF	Association with HSV-2 IgG			R^2 (%)	*F*-statistic
							Beta	SE	*P*-value		
rs10888851	1	54,893,163	SSBP3	G	C	0.11	-0.24	0.05	3.27×10^{-6}	10.12	23.20
rs10782620	1	76,865,351	ST6GALNAC3	G	T	0.40	0.16	0.03	2.60×10^{-6}	10.32	23.70
rs355547	2	142,431,131	LRP1B	C	T	0.39	0.17	0.04	2.00×10^{-6}	10.54	24.28
rs35213774	2	145,157,858	ZEB2	G	A	0.11	0.27	0.05	1.10×10^{-6}	11.05	25.60
rs10174926	2	1,789,294	MYT1L	C	T	0.13	-0.24	0.05	9.72×10^{-7}	11.16	25.89
rs72804080	2	59,358,659	LINC01122	G	A	0.13	0.26	0.05	1.92×10^{-7}	12.54	29.54
rs10964023	9	19,170,162	PLIN2	T	G	0.19	-0.19	0.04	3.58×10^{-6}	10.05	23.00
rs10790877	11	127,242,674	LOC101929497	G	A	0.53	0.16	0.03	7.82×10^{-7}	11.35	26.37

Abbreviations: SNP, single nucleotide polymorphism; CHR, chromosome; EAF, effect allele frequency; SE, standard error.

Notes: Proportion of variance explained by individual SNP was calculated as $R^2 = 2*\beta^2*EAF*(1-EAF)/(2*\beta^2*EAF*(1-EAF) + 2*SE^2*N*EAF*(1-EAF))$, where sample size was denoted by N = 208 (seropositive individuals recruited in the *Milieu Intérieur* cohort); *F*-statistic was computed using the formula $F = R^2*(N-2)/(1-R^2)$, where $F < 10$ indicated a weak instrument variant. The aforementioned equations were given by H. Shim, *et al., PLoS One* **10**, e0120758 (2015).

Supplementary Table 5. Summary-level statistics from the genome-wide association study of multiple sclerosis

Exposure	SNP	CHR	Position (GRCh37)	Nearest Gene	EA/OA	Association with MS		
						Beta	SE	*P*-value
Self-reported HSV	rs144709583	1	165,967,856	FAM78B	T/C	0.104	0.230	0.652
Self-reported HSV	rs900978	2	193,321,821	TMEFF2	T/C	-0.008	0.017	0.641
Self-reported HSV	rs9289557	3	138,071,604	MRAS	T/C	-0.047	0.033	0.159
Self-reported HSV	rs146985296	6	52,152,875	MCM3	C/G	-0.096	0.074	0.193
Self-reported HSV	rs34264769	8	2,639,919	CSMD1	A/C	-0.004	0.033	0.897
Self-reported HSV	rs12550275	8	68,909,451	PREX2	T/C	0.012	0.029	0.692
Self-reported HSV	rs4885004	13	73,093,913	MZT1	G/A	-0.021	0.020	0.284
Self-reported HSV	rs9797556	19	46,500,735	CCDC61	G/A	0.003	0.017	0.853
Self-reported HSV	**rs11703494***	22	50,548,214	MOV10L1	T/C	0.003	0.017	0.853
HSV-1 IgG	rs3132935	6	32,171,075	NOTCH4	G/A	-0.125	0.021	**3.40×10^{-9}**
HSV-1 IgG	rs1738233	6	38,766,488	DNAH8	G/A	0.002	0.017	0.914
HSV-1 IgG	rs58599785	6	139,865,470	LOC645434	G/A	0.052	0.024	0.027
HSV-1 IgG	rs10977313	9	8,828,849	PTPRD	T/C	0.018	0.026	0.490

HSV-2 IgG	rs10888851	1	54,893,163	SSBP3	G/C	-0.004	0.026	0.867
HSV-2 IgG	rs10782620	1	76,865,351	ST6GALNAC3	G/T	0.046	0.017	0.006
HSV-2 IgG	rs355547	2	142,431,131	LRP1B	C/T	0.009	0.017	0.595
HSV-2 IgG	rs35213774	2	145,157,858	ZEB2	G/A	-0.002	0.025	0.923
HSV-2 IgG	rs10174926	2	1,789,294	MYT1L	C/T	-0.015	0.029	0.619
HSV-2 IgG	rs72804080	2	59,358,659	LINC01122	G/A	0.014	0.027	0.606
HSV-2 IgG	rs10964023	9	19,170,162	PLIN2	T/G	0.031	0.026	0.238
HSV-2 IgG	rs10790877	11	127,242,674	LOC101929497	G/A	-0.007	0.017	0.687

Abbreviations: SNP, single nucleotide polymorphism; CHR, chromosome; EA/OA, effect allele/other allele; SE, standard error.

Notes: ***** Rs11703494 (chr22:50548214) was used as a proxy for rs200470549 (chr22:50548270) which were in linkage disequilibrium (D' = 1.0, $r^2 = 0.3601$, $p < 0.0001$), where rs200470549(A) allele was correlated with rs11703494(T) allele.
